# Up-regulation of EMT-related gene VCAN by NPM1 mutant-driven TGF-β/cPML signalling promotes leukemia cell invasion

**DOI:** 10.7150/jca.30223

**Published:** 2019-10-21

**Authors:** Liyuan Yang, Lu Wang, Zailin Yang, Hongjun Jin, Qin Zou, Qian Zhan, Yuting Tang, Yao Tao, Li Lei, Yipei Jing, Xueke Jiang, Ling Zhang

**Affiliations:** 1 Key Laboratory of Laboratory Medical Diagnostics Designated by the Ministry of Education, School of Laboratory Medicine, Chongqing Medical University, Chongqing, China.; 2 Center for Hematology, Southwest Hospital, Third Military Medical University, Chongqing, China.; 3 The Center for Clinical Molecular Medical detection, The First Affiliated Hospital of Chongqing Medical University, Chongqing, China.

**Keywords:** acute myeloid leukemia, nucleophosmin, gene mutation, epithelial-mesenchymal transition, versican, cell invasion

## Abstract

Acute myeloid leukemia (AML) with mutated *nucleophosmin* (*NPM1*) is acknowledged as a distinct leukemia entity in the 2016 updated World Health Organization (WHO) classification. NPM1-mutated AML patients are correlated with higher extramedullary involvement. Epithelial-mesenchymal transition (EMT) is one of the key steps which cause distant metastasis in tumor. However, whether EMT-related programs contribute to cell invasion in NPM1-mutated AML remains unclear. In this study, we identified the EMT-related gene *versican* (*VCAN*) in NPM1-mutated AML across three patient datasets. Further experiments validated the elevated VCAN expression in NPM1-mutated AML primary blasts and OCI-AML3 cells with NPM1 mutation. Mechanistic studies revealed that increased VCAN expression was at least partially regulated by NPM1 mutant via TGF-β/cPML/Smad signalling. Functional evaluations showed that silencing VCAN by shRNA significantly suppressed cell migration and invasion capacity, whereas increased VCAN by overexpressing NPM1-mA enhanced migration and invasion ability of leukemia cells. Finally, we found that high expression of VCAN was associated with poor prognosis in AML patients. These findings provide insights into the involvement of EMT-related gene *VCAN* in the pathogenesis of NPM1-mutated leukemia, which suggests that VCAN is an attractive target for novel diagnostic and therapeutic strategies in NPM1-mutated AML.

## Introduction

Acute myeloid leukemia (AML) is the most common acute leukemia in adults. There are large chromosomal translocations as well as mutations in the genes involved in this genetically heterogeneous disease 1, 2. Mutations in *nucleophosmin* (*NPM1*) gene are frequent events in AML, present in 20-30% of cases 3, 4. With unique biological and clinical features, NPM1-mutated AML is recognized as a distinct entity in the 2016 updated World Health Organization (WHO) classification of myeloid neoplasms and acute leukemia 5.

The* NPM1* gene maps to chromosome 5q35 in humans, contains 12 exons. It encodes for a multifunctional nucleocytoplasmic shuttling protein that is localized mainly in the nucleolus. *NPM1* mutations generate NPM1 mutants that localize aberrantly in the leukemic cell cytoplasm 6. To date, more than 50 different mutations located within exon 12 (a few located within exon 11) of the *NPM1* gene have been identified 7. The most common NPM1 mutation, named as type A mutation (NPM1-mA), is a duplication of a TCTG tetranucleotide at position 956 to 959 of the *NPM* gene sequence (GenBank accession number NM_002520) and accounts for 70% to 80% of cases. Mutations type B (CATG insertions at position 960) and type D (CCTG insertions at position 960) are observed in about 10% and 5% of NPM1-mutated AML; other mutations are very rare 8. AML with NPM1 mutations has been clinically shown to associate with higher extramedullary involvement frequencies, which were generally responsible for gingival hyperplasia, lymphadenopathy and myeloid sarcoma 8, 9. In addition, there was a unique association between NPM1 mutation status and the presence of leukemia cutis, the infiltration of skin with leukemia cells 10. However, the mechanisms underlying these infiltration activities are not yet fully understood. Our previous experimental data showed that NPM1 mutant promoted migration and invasion of leukemia cells through matrix metalloproteases (MMPs) up-regulation 11. It is interesting to note that the epithelial-mesenchymal transition (EMT), characterized by actin cytoskeleton reorganization, increased expression of MMPs and remodeling of extracellular matrix, plays important roles in cancer invasion and metastasis 12-15. Therefore, further studies are needed to elucidate whether the EMT-like process is involved in the invasion phenotype of NPM1-mutated AML.

EMT is a process through the transdifferentiation of epithelial cells into motile mesenchymal cells and is gradually found to play a vital role in nonepithelial tumors, including hematologic malignancies 16. The hallmarks of the EMT program are loss of epithelial markers, such as E-cadherin and ZO-1, acquisition of mesenchymal markers including vimentin, N-cadherin and fibronectin 15. Intriguingly, it is reported that low expression of *CDH1*, which encodes E-cadherin, was an independent unfavorable prognostic factor in AML patients 17, and Wu et al. observed that overexpression of the vimentin was associated with poor clinical outcome in older patients with AML 18. The involvement of prominent EMT transcription factor families has also been reported in AML. For example, *Twist1*-overexpressing AML patients exhibited a more aggressive clinical phenotype 19. Additionally, *ZEB1* was involved in the invasive behavior of MLL-AF9-induced AML cells 20. These studies supported the concept that EMT gene programs play a role in leukemia. Nevertheless, the association between EMT-related genes and NPM1-mutated AML has not yet been studied.

The reprogramming of gene expression during EMT is initiated and controlled by various signalling pathways that respond to extracellular cues 21. Among these pathways, the transforming growth factor-β (TGF-β) family signalling has a prominent role 22. In canonical TGF-β signalling pathway, TGF-β binds to its receptors and subsequently downstream Sma and Mad related family 2 and 3 (Smad2/3) are phosphorylated. Then activated Smad2/3 interact with Smad4 and translocate to the nucleus, which results in the activation of EMT-related genes at transcription levels 23. Several studies have demonstrated that cytoplasmic promyelocytic leukaemia (cPML) appears to favor the phosphorylation of Smad2/3 and acts as an essential modulator of TGF-β signalling 24. Importantly, the cPML could promote TGF-β-associated EMT and invasion in prostate cancer 25. Recently, aberrant cytoplasmic localization of PML was observed in NPM1-mutated AML cells 26, and the PML delocalization was mediated by interacting with NPM1 mutant 27, which suggests that NPM1 mutant might be implicated in the regulation of EMT-related genes expression via cPML in AML.

In this study, we firstly identified the dysregulated EMT-related genes in NPM1-mutated AML from three publicly available datasets, and validated *versican* (*VCAN*) as a key up-regulated target gene. We then observed that the increased VCAN was at least partially regulated by NPM1-mA through TGF-β/cPML/Smad signalling. Finally, we found that knockdown of *VCAN* significantly reduced the invasive capacity of leukemia cells, and further discovered that high expression of *VCAN* was associated with poor outcome in AML patients. These findings for the first time provide insights into the involvement of EMT-related gene *VCAN* in the pathogenesis of NPM1-mutated leukemia, which makes this protein an interesting target in leukemia.

## Materials and Methods

### Identification of differentially expressed genes

With the goal of identifying the differentially expressed genes (DEGs), we used three datasets which primarily included AML with or without NPM1 mutations samples: the Cancer Genome Atlas (TCGA) dataset (n = 179), the GSE34860 dataset (n = 79) and the GSE6891 dataset (n = 461). The gene expression data and clinical data for the TCGA were downloaded from the TCGA data portal (https://www.cancergenome.nih.gov/). The gene expression data for the GSE34860 and GSE6891 were downloaded from the Gene Expression Omnibus (GEO) website (http://www.ncbi.nlm.nih.gov/gds/). The DEGs between *NPM1*-mutated and *NPM1*-unmutated cases in each dataset were analyzed by unpaired* t*-test. The Benjamini Hochberg method was used to adjust the raw *p*-values into false discovery rate (FDR). A threshold with *p* < 0.05 and FDR < 0.05 was selected to determine significant differences in gene expression. The counts of overlapping DEGs among the three datasets were visualized in the Venn diagrams.

### Patient samples

The peripheral blood samples of 42 AML patients newly diagnosed, including 14 *NPM1*-mutated cases and 28 *NPM1*-unmutated cases, were obtained from Southwest Hospital of the Third Military Medical University and the First Affiliated Hospital of Chongqing Medical University. Mononuclear cells were enriched by Ficoll gradient purification and were used for VCAN relative expression analyses. Details of the clinical characteristics of patients are provided in Table 1.

### Cell culture

Human myeloid leukemia cell lines KG1a and THP-1 were obtained from the American Type Culture Collection (ATCC, MD, USA). Human myeloid leukemia cell line OCI-AML3 (harboring NPM1 mutation type A, NPM1-mA) was obtained from Deutsche Sammlung von Mikroorganismen und Zellkulturen GmbH (DSMZ, Braunschweig, Germany). The cells were maintained in RPMI-1640 medium (Gibco, MD, USA) supplemented with 10% fetal bovine serum (FBS; Gibco, MD, USA) and 1% penicillin-streptomycin (Beyotime, Shanghai, China). Cultured cells were incubated in a humidity chamber (Thermo Fisher Scientific, MA, USA) containing 5% CO_2_ at 37 ˚C.

### Reverse transcription PCR and quantitative real-time PCR

Total RNA was isolated using TRIzol reagent (Takara, Kyoto, Japan) and transcribed into cDNA using the PrimeScript™ RT reagent Kit (Takara, Kyoto, Japan). Quantitative real-time PCR (qRT-PCR) analysis of VCAN and β-actin were performed on an MJ Mini™ Gradient Thermal Cycler Real-Time PCR machine (Bio-Rad, CA, USA) with the SYBR Green reaction kit (KAPA Biosystems, MA, USA). The relative fold change for gene expression was calculated using 2^-ΔΔCt^ method, with β-actin mRNA as an internal control. Three independent experiments were performed. Details of the primer sequences used are shown in Table 2.

### Immunocytochemistry

The cultured cells were washed with PBS and cytospun on slides for 15 min, then fixed with 4% paraformaldehyde for 20 min and permeabilized with 1% Triton for 20 min at room temperature. Following blocking with 1% bovine serum albumin in PBS for 30 min, cells were immunostained with rabbit monoclonal VCAN antibody (Abcam, USA, 1:200, Cat No: ab177480) overnight at 4 °C. The primary antibody was revealed using the Ventana Optiview DAB (3,3'-Diaminobenzidine) Detection System. The slides were subsequently counterstained with Mayer's hematoxylin and analyzed using a bright field microscope.

### Western blot

The cultured cells were harvested and lysed in ice-cold RIPA lysis buffer supplemented with protease inhibitor cocktail (Roche, Basel, Switzerland). The protein concentration of cell lysates was determined by BCA Protein Assay Kit (Beyotime, Shanghai, China). Equal amounts of protein (40 μg) were separated by SDS-PAGE and transferred to polyvinylidene difluoride (PVDF) membranes. The specific primary antibodies used in this study were: VCAN (1:200; Abcam, USA, Cat No: ab177480), p-Smad2/3 (1:1000; Cell Signaling Technology, USA, Cat No: 8828), Smad2/3 (1:1000; Cell Signaling Technology, USA, Cat No: 8685), PML (1:1000; Abcam, USA, Cat No: ab200200), rabbit polyclonal mutant NPM1 antibody (1:2000; Thermo Fisher, USA, Cat No: PA1-46356), rabbit polyclonal antibody β-actin (1:1000; Proteintech, USA, Cat No: 20536-1-AP), anti-rabbit secondary antibody (1:4000; Proteintech, USA, Cat No: SA00001-2), and visualization of the products was done using an ECL detection kit (Millipore, MA, USA). The proteins were quantified using image software and normalized against β-actin.

### LY364947 and LMB treatment

The TGF-β signalling inhibitor LY364947 (Selleckchem, TX, USA) was used to treat OCI-AML3 cells with different concentrations (0, 20, 30, 40, 50 μM) for 3 h. For inhibition of cytoplasmic PML levels, leptomycin B (LMB; Beyotime, Shanghai, China) was used to treat OCI-AML3 cells with 20 nM for 6 h. The treated cells were harvested for qRT-PCR and western blot assay.

### Cell transfection and infection

Stably *PML*-silenced (shPML) or *NPM1*-silenced (shNPM1) OCI-AML3 cells were generated as previously described by us 27. Plasmids encoding Flag-NPM1-wt, Flag-NPM1-mA and empty vector were obtained from Dr. C.J. Sherr (Genetics and Tumor Cell Biology, St, Jude Children's Research Hospital, Memphis, USA). Plasmids encoding Xp-cPML-WT were obtained from Dr. H.K. Lin (Department of Molecular and Cellular Oncology, The University of Texas MD Anderson Cancer Center, Houston, Texas, USA), which was designated as “cPML” groups. All transfection experiments were conducted using Neofect™ DNA transfection reagent Kit (Neofect Biotechnologies, Beijing, China) according to manufacturer's instructions, after 48 h of transfection, the cells were collected for qRT-PCR and western blot analyses. The shRNA targeting VCAN (5′-CCGGGCCACAGTTATTCCAGAGATTCTCGAGAATCTCTGGAATAACTGTGGCTTTTTG-3′, the shRNA sequence is derived from Said et al*.* 28) and scramble lentiviral vectors were purchased from Genechem (Shanghai, China), respectively. OCI-AML3 cells were infected with shRNA lentivirus targeting VCAN for 48 h in the presence of 5 μg/mL polybrene (Sigma, CA, USA), following 2 μg/mL puromycin selection for 7 days (Sigma, CA, USA), respectively. The puromycin-resistant cells were isolated and propagated for use in the experiments.

### Transient siRNA transfection

The siRNAs targeting VCAN-V1 were chemically synthesized by GenePharma (Shanghai, China). The following siRNA sequences which derived from Onken et al. 29 and Zhang et al. 30 were selected: siV1-1 (sense: 5'-GGGAGUUCUUCGAUUCCAA-3'; antisense: 5'-UUGGAAUCGAAGAACUCCC-3'); siV1-2 (sense: 5'-GAGGCUGGAACUGUUAUUA-3'; antisense: 5'-UAAUAACAGUUCCAGCCUC-3'). The KG1a cells were transfected with siRNA using the GP-siRNA-Mate Plus transfection Reagent (GenePharma, Shanghai, China) according to the manufacturer's instructions. After 36 ~ 48 h of transfection, the cells were collected for qRT-PCR or western blot analysis.

### Cell viability assay

Cell viability was determined using Cell Counting Kit-8 (CCK-8; Dojindo Laboratories, Kumamoto, Japan) according to the manufacturer's instruction. In Brief, OCI-AML3 and KG1a cells were seed at 1×10^3^ ~ 1.5×10^3^ cells in a 96-well plate for 0 h, 12 h, 24 h, 36 h, 48 h. At the indicated time point, cells were treated with CCK-8 at 10 μl/well at 37 °C for 3 h, and the number of cells per well was determined by measuring absorbance at 450 nm.

### Cell migration and invasion assay

A total of 5×10^4^ cells were seeded into the upper chamber of a transwell insert (8 μm pore size; Corning, NY, USA) in 200 μl serum-free RPMI 1640. The upper chamber was then placed into the lower chamber filled with 500 μl RPMI 1640 containing 15% FBS. After cultured at 37 °C for 24 h, the cells that had migrated to the lower chambers were counted using an inverted microscope. The invasion assay was performed in the same manner as the migration assay except each insert was pre-coated with 50 μl Matrigel (BD Biosciences, CA, USA).

### Survival analysis

Gene expression levels and clinical survival information of 166 AML patients, including 47 patients harboring NPM1 mutations, were retrieved from TCGA dataset. All patients were stratified by VCAN expression levels, to categorize patients into either a high cohort (≥ 13.3046) or low cohort (0 to 13.3046). The Kaplan-Meier method was used to analyze the overall survival.

### Statistical analysis

All data were derived from three independent experiments. Student's *t*-test and one-way analyses of variance (ANOVA) were applied to calculate the statistical significance of biological replicate experiments. SPSS (version 17.0; IBM Corporation) and Prism (version 5.0; GraphPad Software) software programs were used to conduct the statistical tests. The Kaplan-Meier estimation and the log-rank test were used to compare the survival difference. *p* < 0.05 was considered statistically significant.

## Results

### Identification of the EMT-related genes in NPM1-mutated AML

**Figure 1:**
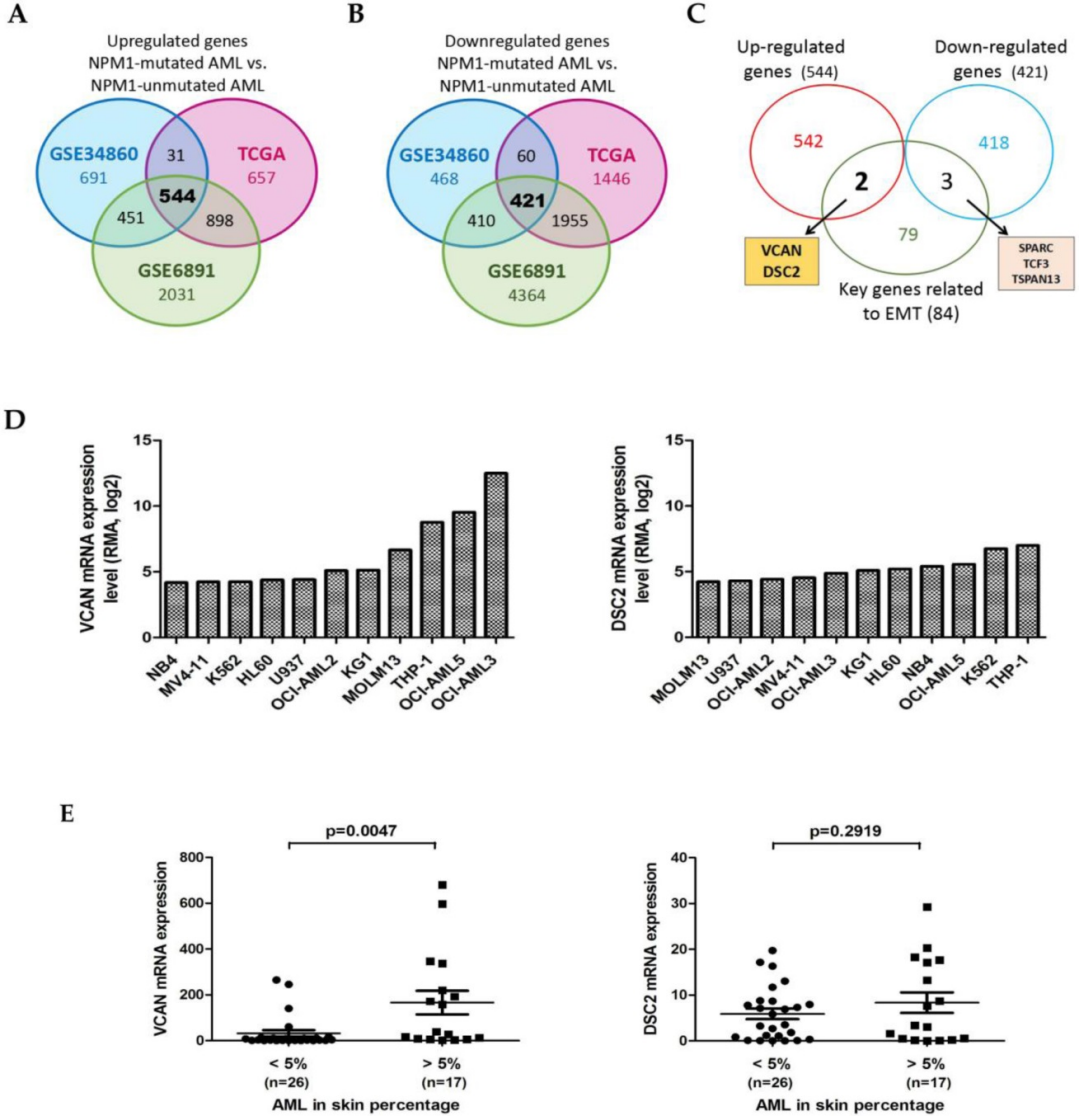
** Identification of the EMT-related genes in NPM1-mutated AML.** (A) Venn diagram showing the overlap among the up-regulated genes (NPM1-mutated AML vs NPM1-unmutated AML) identified from three datasets (TCGA, GSE34860, and GSE6891). (B) Venn diagram showing the overlap among the down-regulated genes (NPM1-mutated AML vs NPM1-unmutated AML) identified from three datasets (TCGA, GSE34860, and GSE6891). (C) Venn diagram showing the overlap among the up-regulated genes, the down-regulated genes and 84 key genes related to EMT. Arrows point to the corresponding lists of overlapping genes. (D) The *VCAN* and *DSC2* mRNA levels in leukemia-lymphoma cell lines derived from the CCLE database. (E) The *VCAN* and *DSC2* mRNA levels in primary human AML samples with different percentage of skin infiltration.

To identify the dysregulated EMT-related genes in NPM1-mutated AML, we firstly determined the differentially expressed genes (DEGs) between AML with or without NPM1 mutation using three publicly available datasets: the GSE34860 dataset (n = 79), the Cancer Genome Atlas (TCGA) dataset (n = 179), and the GSE6891 dataset (n = 461). Comparison of the three resultant of DEGs showed that a total of 544 up-regulated genes and 421 down-regulated genes were present in all three datasets (Figure 1A and 1B). We then combined these candidate genes and 84 key genes related to EMT 31, and screened out 5 dysregulated EMT-related genes including 2 up-regulated genes and 3 down-regulated genes (Figure 1C). In this study, we focused on the up-regulated genes: *VCAN* and *DSC2*. Next, we downloaded the RNA-seq data of *VCAN* and *DSC2* genes in a panel of leukemia-lymphoma cell lines from the website of the Cancer Cell Line Encyclopedia (CCLE, https://portals.broadinstitute.org/ccle/home), and detected higher *VCAN* expression in NPM1-mA positive OCI-AML3 cells (Figure 1D). Given that skin infiltration is a feature of the extramedullary infiltration phenomena, we further investigated whether the *VCAN* and *DSC2* expression were associated with this infiltration activity. The available clinical data for the 43 AML samples with skin infiltration from the TCGA dataset were divided into the two groups with “high level” (> 5%) or “low level” (< 5%) skin percentage. We found that the expression of *VCAN* was increased in the patient group with “high level” skin infiltration (*p* = 0.0047), whereas there was no statistical difference in the expression of *DSC2* between the two groups (*p* = 0.2919) (Figure 1E). Therefore, we selected VCAN for further investigation.

### Detection of VCAN expression levels in NPM1-mutated leukemia cells

**Figure 2:**
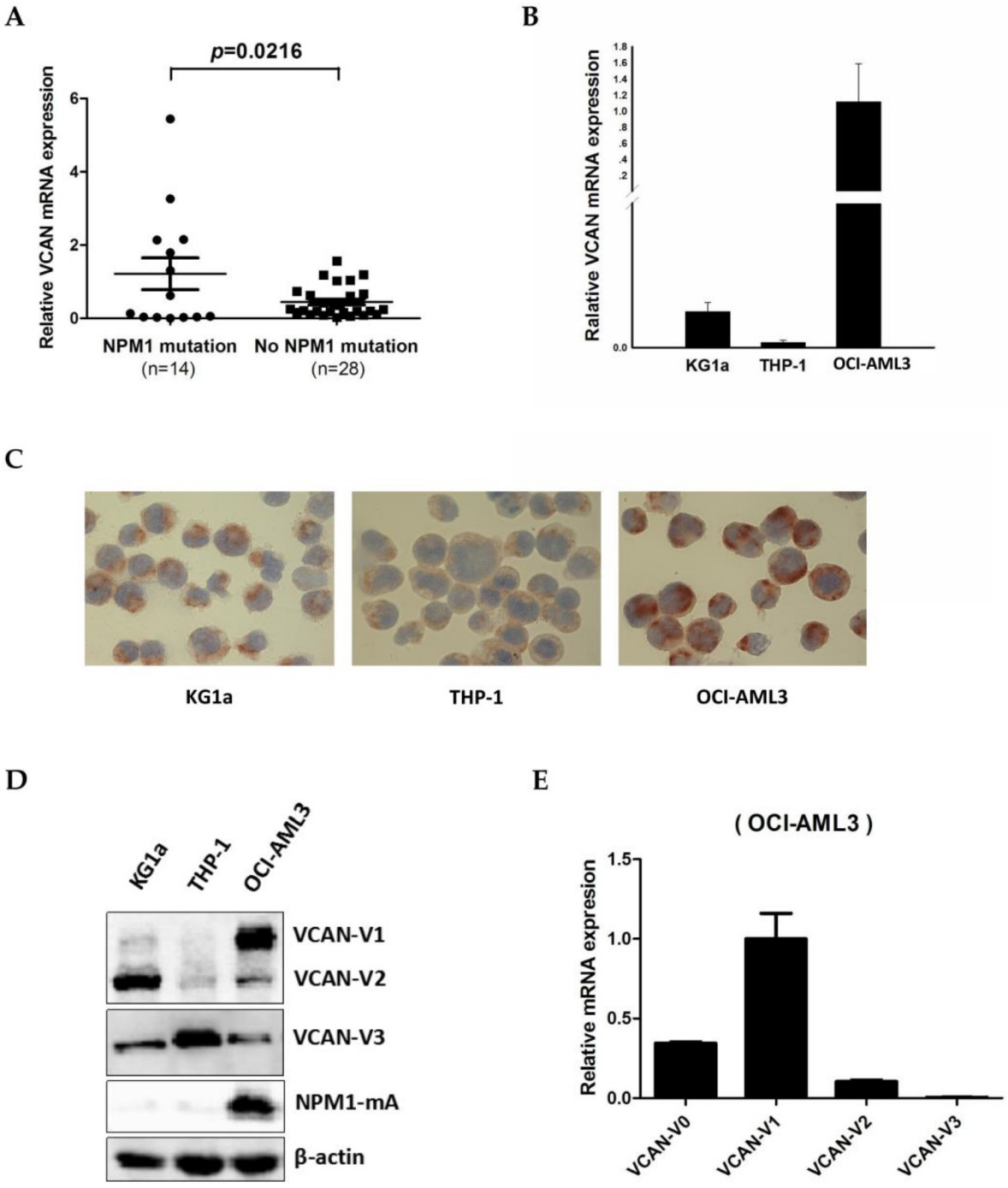
** The expression levels of VCAN in NPM1-mutated leukemia cells.** (A) Expression levels of total *VCAN* mRNA in primary NPM1-mutated AML samples (n = 14) were assessed by qRT-PCR and compared to NPM1-unmutated AMLs (n = 28). (B) qRT-PCR showing the total *VCAN* mRNA levels in three myeloid leukemia cell lines. (C) Representative results of total VCAN (brown) detected by immunocytochemistry staining (DAB, ×400) in OCI-AML3 versus KG1a and THP-1 cells. (D) Western blot showing the VCAN isoforms protein levels in three AML cell lines. (E) qRT-PCR showing the *VCAN* isoforms mRNA levels in OCI-AML3 cell line. Data were represented as mean ± s.d. of three individual experiments.

To detect the VCAN expression in NPM1-mutated AML, we firstly measured the expression of *VCAN* mRNA levels in 42 primary AML blasts by quantitative real-time PCR (qRT-PCR). The results showed that the *VCAN* transcript levels were increased among the cases of AML with NPM1 mutations (n = 14), as compared to those without NPM1 mutations (n = 28) (*p* = 0.0216, Figure 2A). Next, we examined VCAN mRNA and protein levels in three human myeloid leukemia cell lines. As an obvious consequence, OCI-AML3 cells with NPM1 mutation had significantly higher *VCAN* mRNA expression than that in KG1a and THP-1 cells without NPM1 mutations (Figure 2B). Accordingly, VCAN protein expression detected by immunocytochemistry staining also appeared to be more prominent in OCI-AML3 cells with the presence of much more brown precipitate (Figure 2C). Furthermore, VCAN protein expression was determined by western blot analysis. Alternative splicing of VCAN concludes four common isoforms, VCAN-V0, VCAN-V1, VCAN-V2 and VCAN-V3 32. Our results revealed that the antibody applied in this experiment detected three isoforms: VCAN-V1, VCAN-V2 and VCAN-V3, and VCAN-V1 was the highest expressed isoform in OCI-AML3 cells (Figure 2D). In support, the qRT-PCR analysis detected the four isoforms and also showed the obviously increased expression of *VCAN-V1* at the level of transcript (Figure 2E). These results demonstrated that VCAN-V1 was highly expressed in NPM1-mutated AML.

### VCAN-V1 is up-regulated by NPM1-mA via TGF-β/cPML/Smad signalling in OCI-AML3 cells

**Figure 3:**
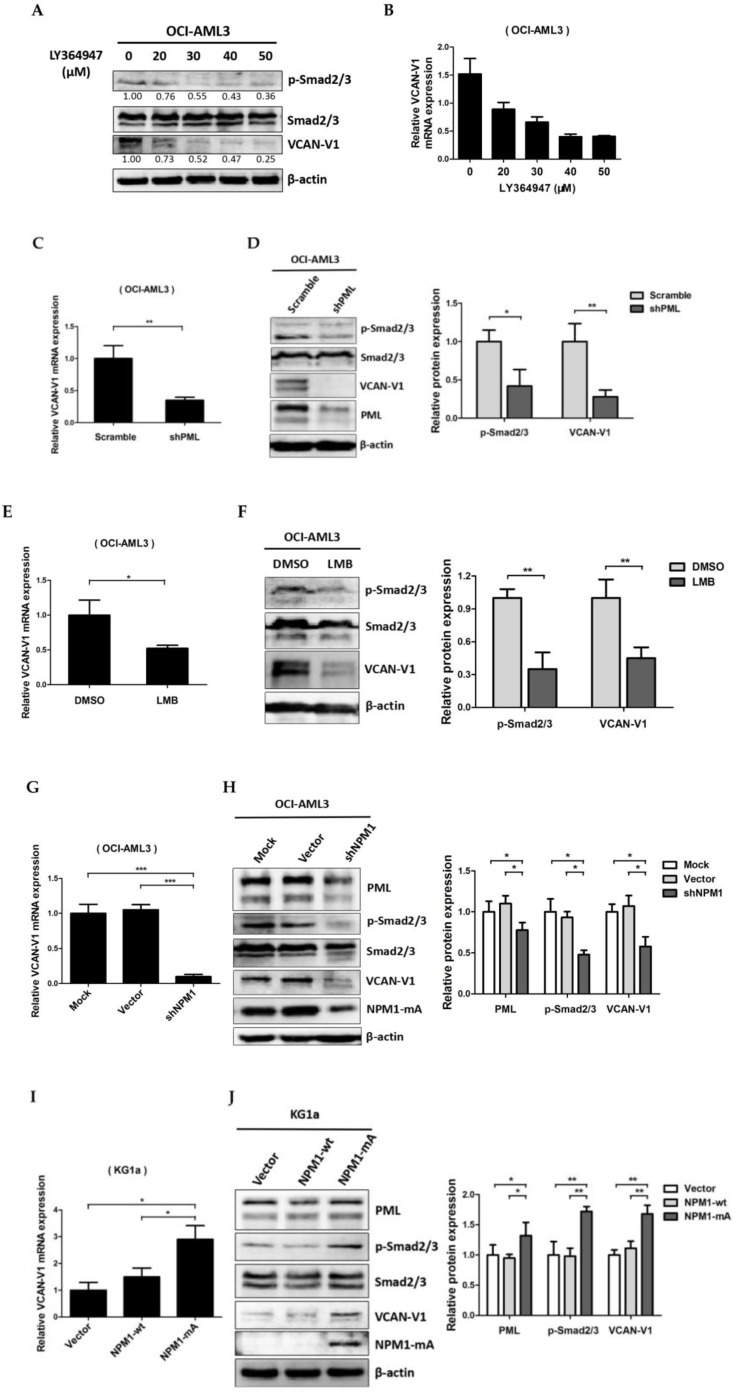

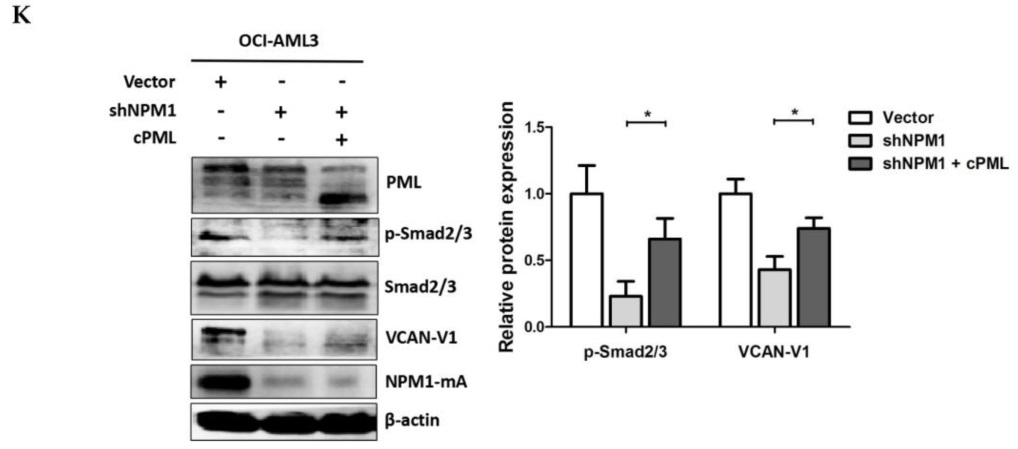
** VCAN-V1 is up-regulated by NPM1-mA via TGF-β/cPML/Smad signalling in OCI-AML3 cells.** (A) Western blot analysis of p-Smad2/3, Smad2/3 and VCAN-V1, (B) qRT-PCR analysis of *VCAN-V1* mRNA expression from OCI-AML3 cells treated with LY364947. (C) qRT-PCR analysis of *VCAN-V1* mRNA expression, (D) western blot analysis of p-Smad2/3, Smad2/3, VCAN-V1 and PML from the PML-silenced OCI-AML3 cells. (E) qRT-PCR analysis of *VCAN-V1* mRNA expression, (F) western blot analysis of p-Smad2/3, Smad2/3, VCAN-V1 from the OCI-AML3 cells treated with 20 nM LMB. (G) qRT-PCR analysis of *VCAN-V1* mRNA expression, (H) western blot analysis of PML, p-Samd2/3, Smad2/3, VCAN-V1 and NPM1-mA from the NPM1-silenced OCI-AML3 cells. (I) qRT-PCR analysis of *VCAN-V1* mRNA expression, (J) western blot analysis of PML, p-Samd2/3, Smad2/3, VCAN-V1 and NPM1-mA from the KG1a cells transduced with the plasmids expressing NPM1-wt or NPM1-mA. (K) Western blot analysis of PML, p-Smad2/3, Smad2/3, VCAN-V1, and NPM1-mA from the NPM1-silenced OCI-AML3 cells transfected with plasmids expressing cPML. Proteins were quantified using image software and normalized against β-actin. Data were represented as mean ± s.d. of three individual experiments. * *p* < 0.05, ** *p* < 0.01, *** *p* < 0.001.

To evaluate the molecular mechanism underlying VCAN-V1 up-regulation in leukemia cells with NPM1 mutation. Firstly, we used TGF-β signalling inhibitor (LY364947) to treat the OCI-AML3 cells. As an obvious result, the levels of phosphorylated Smad2/3 (p-Smad2/3) were decreased in a dose-dependent manner (Figure. 3A). Notably, the VCAN-V1 mRNA and protein levels were considerably reduced (Figure 3A and 3B). Given that the cPML is an essential modulator of TGF-β signalling 24, 25, we then evaluated the effect of cPML in the regulation of VCAN expression. From the data in Figure 3C and 3D, it is apparent that deletion of PML considerably reduced VCAN-V1 mRNA and protein levels. Our newly published report showed that treatment with leptomycin (LMB) could suppress the cytoplasmic PML levels in OCI-AML3 cells 27. At present, we found that LMB treatment evidently down-regulated VCAN-V1 mRNA and protein expression (Figure 3E and 3F). Considering that the delocalization of PML in NPM1-mutated AML was mediated by interacting with NPM1-mA 27, we examined the impact of mutated NPM1 on the expression of VCAN. The results revealed that loss of NPM1-mA obviously declined the VCAN-V1 mRNA and protein levels in OCI-AML3 cells (Figure 3G and 3H). On the contrary, ectopic overexpression of NPM1-mA increased the VCAN-V1 mRNA and protein levels in KG1a cells (Figure 3I and 3J). Finally, we tested the role of cPML in NPM1-mA-mediated VCAN expression by a rescue experiment. The results demonstrated that the decreased p-Smad2/3 and VCAN-V1 expression induced by NPM1 silencing was rescued by the introduction of exogenous cPML (Figure 3K). Taken together, these results demonstrated that high levels of VCAN-V1 were at least partially caused by the NPM1-mA via TGF-β/cPML/Smad signalling in OCI-AML3 cells.

### VCAN-V1 knockdown attenuates migration and invasion capacity of OCI-AML3 cells

**Figure 4:**
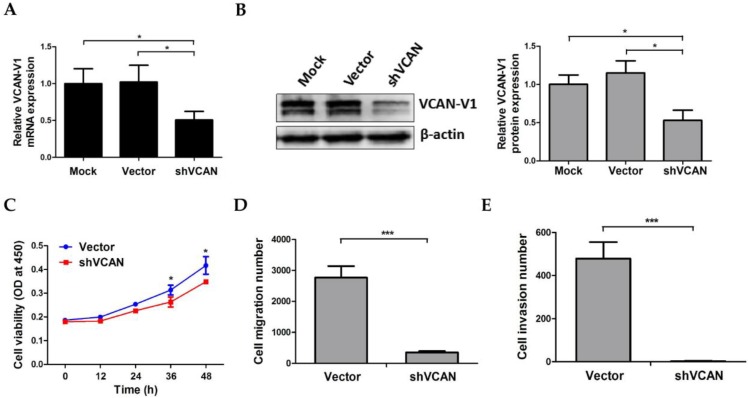
** Effect of VCAN-V1 knockdown on OCI-AML3 cells migration and invasion capacity.** (A) qRT-PCR of *VCAN-V1* mRNA expression, (B) western blot analysis of VCAN-V1 expression from the OCI-AML3 cells infected with shRNA lentivirus targeting VCAN. Proteins were quantified using image software and normalized against β-actin. (C) CCK-8 analyzed the effect of VCAN-V1 knockdown on cell proliferation in OCI-AML3 cells. (D) Transwell chambers inserted in 24-well tissue culture plates were used for detecting cell migration. The number of migration cells in the shVCAN group was significantly lower than that in the vector group. (E) Transwell chambers with a Matrigel coating were used to test the invasion ability of the cells. The number of invasive cells in the shVCAN group was significantly lower than that in the vector group. Data were represented as mean ± s.d. of three individual experiments. * *p* < 0.05, *** *p* < 0.001.

To explore the functional significance of VCAN-V1 upregulation on cell migration and invasion properties in NPM1-mutated leukemia, OCI-AML3 cells were stably infected with shRNA lentivirus targeting VCAN and the decreased levels of VCAN-V1 mRNA and protein were successfully confirmed (Figure 4A and 4B). We then examined the change in cell proliferation and insured that VCAN-V1 silencing would not influence OCI-AML3 cells growth within 24 h as compared to the vector group, so as to ensure that the migration and invasion assay was not affected by the cell growth number (Figure 4C). The migration assay indicated that the cell number in the lower chambers of the shVCAN group was notably less than that in the vector group (*p* < 0.001, Figure 4D). In addition, Matrigel invasion assay demonstrated that the number of invasive cells was significantly decreased in shVCAN group compared with the vector group (*p* < 0.001, Figure 4E). These results indicated that VCAN-V1 knockdown could attenuate OCI-AML3 cells migration and invasion capacity.

### Increased VCAN-V1 by overexpressing NPM1-mA enhances migration and invasion ability of KG1a cells

**Figure 5:**
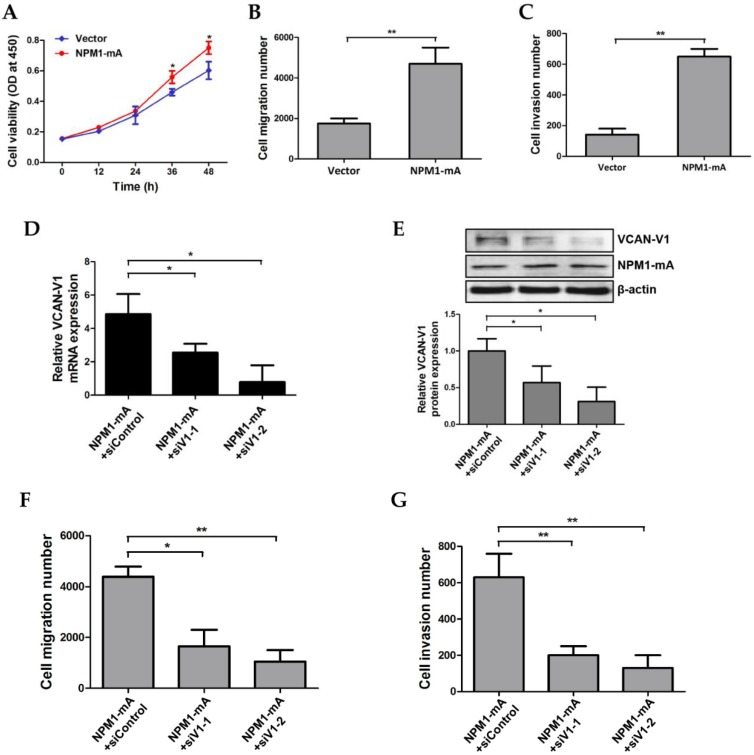
** Increased VCAN-V1 by overexpressing NPM1-mA enhances migration and invasion ability of KG1a cells.** (A) CCK-8 analysis of cell proliferation activity in KG1a cells overexpressed with NPM1-mA. (B) The number of migration cells in the NPM1-mA group was significantly higher than that in the vector group. (C) The number of invasive cells in the NPM1-mA group was higher than that in the vector group. (D) qRT-PCR of *VCAN-V1* mRNA expression, (E) western blot analysis of VCAN-V1 protein expression in NPM1-mA-enforced KG1a cells transfected with siRNAs targeting VCAN-V1. (F) Down-regulation of VCAN-V1 inhibited NPM1-mA-induced cell migration in KG1a cells. (G) Down-regulation of VCAN-V1 inhibited NPM1-mA-induced cell invasion in KG1a cells. Data were represented as mean ± s.d. of three individual experiments. * *p* < 0.05, ** *p* < 0.01.

To further elucidate the effects of VCAN-V1 on cell migration and invasion capacity in leukemia, cell migration and invasion were measured in KG1a cells overexpressed with NPM1-mA that leads to increased VCAN-V1. Given that cell proliferation had no significant differences between the NPM1-mA group and vector group within 24 h (Figure 5A), the migration assay was performed and showed that NPM1-mA overexpression significantly increased the number of migration cells (*p* < 0.01, Figure 5B). In addition, the Matrigel invasion assay revealed that overexpression of NPM1-mA enhanced KG1a cells invasion ability (*p* < 0.01, Figure 5C). Furthermore, a rescue assay was conducted to verify the impact of VCAN-V1 on NPM1-mA-enforced KG1a cells. As shown in Figure 5D and 5E, elevated VCAN-V1 levels induced by the introduction of exogenous NPM1-mA were down-regulated by siRNAs (siV1-1, siV1-2). Importantly, down-regulation of VCAN-V1 inhibited NPM1-mA-induced cell migration and invasion in KG1a cells (Figure 5F and 5G). These results indicated that increased VCAN-V1 by overexpressing NPM1-mA could enhance migration and invasion ability of KG1a cells.

### High expression of VCAN is associated with poor survival outcome in AML

**Figure 6:**
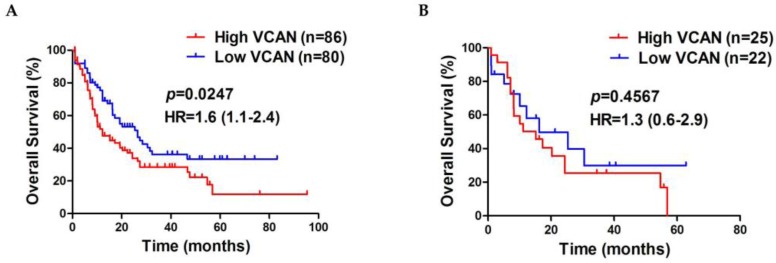
** High expression of VCAN is associated with poor survival outcome in AML.** Survival analysis was performed using Kaplan-Meier curves and the log-rank test to estimate the survival probability. (A) Survival analysis based on total *VCAN* gene expression in TCGA primary AML samples. (B) Survival analysis based on total *VCAN* gene expression in TCGA primary NPM1-mutated AML samples.

There is a growing body of evidence that raised levels of VCAN are strongly associated with poor prognosis of patients in a wide range of malignant tumors 33, 34. To determine the clinical importance of VCAN in AML, we assessed the prognostic value of VCAN using the dataset of TCGA, for which constituted of 166 AML cases with available clinical data and had up to 95 months of follow up. The results showed that a higher VCAN gene expression predicted shorter overall survival in AML cases compared to a lower VCAN gene expression (median, 12.17 vs 26.40 months; *p* = 0.0247, Figure 6A), and high VCAN expression had a 1.1 - 2.4 fold higher hazard ratio (HR). Moreover, we observed that NPM1-mutated AML cases with high VCAN gene expression were associated with a short survival time compared to low VCAN expression cohort, whereas there was no statistical difference (median, 15.17 vs 16.20 months; *p* = 0.4567, Figure 6B). Collectively, these clinical data from TCGA dataset indicated that high expression of VCAN showed a trend towards poor prognosis in AML cases.

## Discussion

AML with NPM1 mutation is significantly related to higher extramedullary involvement frequencies. However, the mechanisms involved in this phenomenon have not been fully elucidated. Herein, we identified the dysregulated EMT-related genes in NPM1-mutated AML and validated that *VCAN* was highly expressed. We also demonstrated that the VCAN expression levels were at least partially up-regulated by NPM1-mA via TGF-β/cPML/Smad signalling. Moreover, high expression of VCAN played an important role in leukemia cell migration and invasion, and was potentially associated with poor overall survival of AML patients.

The EMT process is increasingly recognized for playing a key role in the progression, dissemination of solid tumors 35. It is worth noting that EMT-related modulators implicated in the regulation of malignant phenotype in leukemia 36, 37. In this study, we explored whether EMT-related genes were involved in NPM1-mutated AML. Using the expression profile data analysis, we screened out the up-regulated genes (*VCAN* and *DSC2*) based on overlap among three individual datasets. We then revealed that NPM1-mA positive OCI-AML3 cells exhibited the highest VCAN expression among the human leukemia-lymphoma cell lines in the CCLE database, whereas there was no significant difference in DSC2 expression among these cell lines. Furthermore, we investigated whether the VCAN and DSC2 expression was associated with extramedullary involvement in primary human AML, and found that the expression of VCAN but not DSC2 was correlated with skin infiltration in the analysis of TCGA dataset. Given the above findings, we focused on the VCAN gene for further study. It has been known that VCAN is an aggregating chondroitin sulfate proteoglycan, one of the major components of extracellular matrix 38, 39, which plays a role in the EMT process 40-42. Elevated levels of VCAN have been reported in many types of cancers to date, including prostate cancer 43, melanomas 44, breast 45, ovarian cancer 46 and colorectal cancer 47. Recent data also demonstrated that the AML patients showed significantly increased plasma and leukocytes VCAN levels when compared with the healthy subjects and ALL patients 48. In the present study, we validated the up-regulation expression of VCAN in NPM1-mutated AML primary blasts. Additionally, we discovered that OCI-AML3 cells had distinctly higher VCAN expression and harbored the highest expression of VCAN-V1 isoform. Indeed, the unbalanced expression of VCAN isoforms was described in pathological conditions, VCAN-V0 and VCAN-V1 were the predominant isoforms present in cancer tissues 49, 50.

EMT can be naturally induced by several cellular pathways such as TGF-β, Wnt, Sonic Hedgehog (SHH) and Notch signalling. Notably, TGF-β signalling is a well-established pathway that controls target gene transcription during EMT 51. In this study, we determined whether the increased expression of VCAN might be regulated by the TGF-β signalling pathway in NPM1-mutated AML. Firstly, TGF-β/Smad inhibitor LY364947 was used to treat OCI-AML3 cells. We found that the inhibition of TGF-β signalling reduced the VCAN-V1 mRNA and protein expression in a dose-dependent manner. This result is consistent with those of other reports, in which the VCAN excessive production could be abrogated by the addition of a TGF-β-neutralizing antibody or in the presence of TGF-β receptor and SMAD3-specific inhibitors in ovarian cancer associated fibroblasts 52, 53. It is known that cytoplasmic PML (cPML) is an essential modulator of TGF-β signalling 24. Recent outcomes have revealed that abnormal increased cytoplasmic localization of PML appears in NPM1-mutated leukemia cells 26. Then we assessed the role of cPML in the regulation of VCAN-V1 expression. Results confirmed that silenced PML decreased the VCAN-V1 mRNA and protein levels. Of note, our newly published report demonstrated that the specific nuclear export inhibitor LMB could suppress the cytoplasmic PML levels in OCI-AML3 cells. In the current study, LMB was used to treat OCI-AML3 cells and markedly declined the expression of p-Smad2/3 and VCAN-V1. Considering that aberrant cytoplasmic localization of PML was mediated by NPM1-mA in NPM1-mutated leukemia cells 27, we next investigated the effects of NPM1-mA on VCAN-V1 expression. The results demonstrated that NPM1-mA knockdown decreased PML levels and further inhibited VCAN-V1 expression, whereas forced expression of NPM1-mA had the opposite effect. Finally, we performed a rescue assay and discovered that ectopic expression of cPML could reverse the reduction of VCAN-V1 expression mediated by NPM1 depletion. These observations indicate that VCAN-V1 is at least partially up-regulated by NPM1-mA-driven TGF-β/cPML/Smad signalling. Certainly, in addition to TGF-β signalling, VCAN was also found to be a target gene of Wnt signalling and SHH signalling 54. Further studies are warranted to elucidate whether other signalling pathways might involve in the regulation of VCAN expression in NPM1-mutated AML.

EMT is a crucial mechanism for effective metastatic dissemination and EMT-related factors are associated with migration and invasion capability of tumor cells 36, 55. Here we tested whether VCAN-V1 was involved in the regulation of NPM1-mutated leukemia cells invasion. Results of our functional studies showed that VCAN-V1 depletion significantly inhibited migration and invasion capacity of OCI-AML3 cells. Additionally, up-regulation of VCAN-V1 by overexpressing NPM1-mA significantly enhanced migration and invasion ability of KG1a cells, whereas down-regulation of VCAN-V1 suppressed the NPM1-mA-induced cell migration and invasion in KG1a cells. Previous studies have shown that VCAN-V1 up-regulation facilitated skin homing of lymphoid cells by promoting their migration in Sézary cells 50. In addition, Mitsui et al*.* demonstrated that VCAN knockdown inhibited renal carcinoma cells migration and invasion 56. Recently, Zhang et al. reported that elevated levels of VCAN were essential for Snail-mediated breast cancer cell migration and metastasis 57. Indeed, one of the critical effects of VCAN aberration is generating a microenvironment conducive for cancer cell migration and invasion 58. These findings and our data imply that VCAN-V1 might contribute to the invasive potential of NPM1-mutated leukemia cells. In our experiments, OCI-AML3 cell line that is accessible was selected for research. In a future study, we will use another mutant NPM1-expressing IMS-M2 cell line 59 to verify the role of VCAN in cell migration and invasion activity. Meanwhile, the function of VCAN in mouse knock-in models that mimic human NPM1-mutated AML is needed to be further investigated as well.

Finally, we evaluated the clinical significance of VCAN in AML by analyzing the data derived from the TCGA dataset and found that high levels of VCAN delineated a poorer prognosis in AML patients. These results are consistent with those from another report where the overexpression of VCAN is an adverse prognostic factor in MLL-rearranged infant acute lymphoblastic leukaemia 60. In addition, El Kramani et al. has recently shown that VCAN correlated with poor response to induction of chemotherapy in AML cases 48. Herein we further estimated the prognostic value of high expression of VCAN in NPM1-mutated AML patients. Unexpectedly, we found no statistical differences in prognosis between the NPM1-mutated AML cases with high VCAN expression and those with low VCAN expression, despite the fact that patients with high VCAN had a slight downward trend of survival time. These results indicated that the elevated expression of VCAN alone maybe not sufficient to predict prognosis in NPM1-mutated AML. In fact, there are many other genetic aberrations (e.g., FLT3-ITD and DNMT3A) correlated to prognosis in NPM1-mutated AML 61, 62. Additionally, we recently discovered that NPM1-mutated patients with high INPP4B expression tended to have shorter survival outcome 63. Therefore, it may be more effective to evaluate NPM1-mutated AML patients clinical prognosis by multivariate analysis.

**Figure 7:**
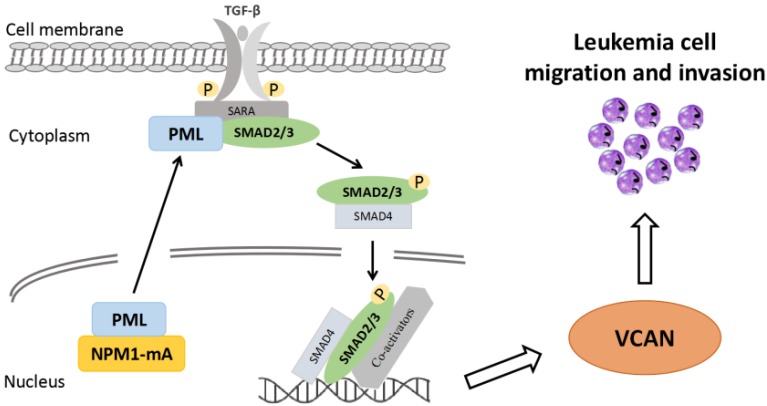
**Schematic diagram describing the functional significance of EMT-related gene *VCAN* in the NPM1-mutated leukemia cells.** The EMT-related gene *VCAN* is highly expressed in NPM1-mutated AML and the expression of VCAN is partially up-regulated by NPM1-mA via TGF-β/cPML/Smad signaling. Furthermore, high VCAN expression promotes leukemia cell migration and invasion.

In summary, to our knowledge, this represents the first study to demonstrate an association between high expression of EMT-related gene *VCAN* and leukemia cell invasive potential. Notably, we verify that the high levels of VCAN are at least partially mediated by NPM1-mA-driven TGF-β/cPML/Smad signalling (Figure 7). Moreover, the increased VCAN expression shows a trend towards poor outcome in AML patients. Our results suggest that VCAN might have therapeutic and/or biomarker potential for NPM1-mutated AML, and might be a prognostic biomarker for AML.

## Figures and Tables

**Table 1:** Patients characteristics

Characteristics	Median (range)	No. of cases
**Sex**		
Female		22
Male		20
Total		42
**Median age, y**	55 (26-79)	
Younger than 40y		10
40-60y		18
Older than 60y		14
**Median WBC, ×10^9^/L**	45 (0.3-299)	
**Median platelets, ×10^9^/L**	58.8 (3.0-660.0)	
**FAB classification**		
M1		3
M2		6
M3		7
M4		11
M5		14
unclassified		1
**Karyotype**		
Normal		16
t (8;21)		7
t (15;17)		6
inv. (16)		9
Unknown		4
**Gene mutations**		
*NPM1*		14
*FLT3/ITD*		13
*WT1*		16

Abbreviations*:* AML, acute myeloid leukemia; y, year old; WBC, white blood cell; FAB classification, French-American-British classification, a classification of acute leukemia produced by three-nation joint collaboration.

**Table 2:** Primer sequences used in this study

Genes	Sequence (5'-3')
*VCAN*	F: 5'-CCACGCTTCCTATGTGA-3' R: 5'-TTTCCCACTTTGACTTTATGT-3'
*VCAN-V0*	F: 5'-GCACAAAATTTCACCCTGACATT-3' R: 5'-CTTCTTTAGATTCTGAATCTATTGGATGAC-3'
*VCAN-V1*	F: 5'-CCCAGTGTGGAGGTGGTCTAC-3' R: 5'-CACTCAAATCACTCATTCGACGTT-3'
*VCAN-V2*	F: 5'-TCAGAGAAAATAAGACAGGACCTGATC-3' R: 5'-CATACGTAGGAAGTTTCAGTAGGATAACA- 3'
*VCAN-V3*	F: 5'-CCCTCCCCCTGATAGCAGAT-3' R: 5'-GGCACGGGTTCATTTTGC-3'
*β-actin*	F: 5'-TAGTTGCGTTACACCCTTTCTTG-3' R: 5'-TGCTGTCACCTTCA CCGTTC-3'

Abbreviations*:* F stands for forward; R stands for reverse

## Abbreviations

AML: Acute myeloid leukemia
NPM1: nucleophosmin
WHO: World Health Organization
EMT: Epithelial-mesenchymal transition
VCAN: versican
TGF-β: transforming growth factor-β
Smad2/3: Sma and Mad related family 2 and 3
cPML: cytoplasmic promyelocytic leukaemia
MMPs: matrix metalloproteases
NPM1-mA: NPM1 mutation type A
DEGs: differentially expressed genes
TCGA: The Cancer Genome Atlas
GEO: Gene Expression Omnibus
FDR: false discovery rate
qRT-PCR: quantitative real-time PCR
CCLE: Cancer Cell Line Encyclopedia
p-Smad2/3: phosphorylated Smad2/3
LMB: leptomycin B
